# An Ominous Cause of Headache in a Teenager

**DOI:** 10.7759/cureus.16934

**Published:** 2021-08-06

**Authors:** Dipal Shah, Rachel Sklar, Latha Ganti, Joshua Walker

**Affiliations:** 1 Emergency Medicine, Ocala Regional Medical Center, Ocala, USA; 2 Emergency Medicine, Brown University, Providence, USA; 3 Emergency Medicine, Envision Physician Services, Plantation, USA; 4 Emergency Medicine, University of Central Florida College of Medicine, Orlando, USA; 5 Emergency Medicine, HCA Healthcare Graduate Medical Education Consortium Emergency Medicine Residency Program of Greater Orlando, Olrando, USA

**Keywords:** pineoblastoma, pediatric headache, cephalgia, emergency medicine, brain tumor

## Abstract

We present the case of an adolescent male who presented to the emergency department with headache and vomiting. We discuss the differential diagnosis and the need to maintain a high index of suspicion to avoid missing ominous causes of headache. In this case, the patient had a pineoblastoma, detected on a noncontrast CT scan. The CT scan was done as part of the emergency department workup to evaluate headache accompanied by vomiting in this otherwise healthy teenager.

## Introduction

The pineal gland is a part of the endocrine system located in the brain. It is responsible for melatonin secretion. Pineal parenchymal tumors are tumors of the pineal gland and include pineocytomas, papillary tumors, pineal parenchymal tumors of intermediate differentiation, and pineoblastomas [1]. Pineoblastomas are most commonly diagnosed in children and young adults [2]. On average, the age of onset is 12.6 years [3]. Adult cases comprise less than 10% of overall cases, and adult pineoblastoma cases have been found to require different treatments than pediatric cases [4]. There is a male predominance, particularly among the pediatric population [5].

Patients with pineoblastoma often present with nonspecific headache and vomiting from elevated intracranial pressure, resulting from a buildup of cerebrospinal fluid [6]. The most common symptoms that present in pediatric patients with pineoblastoma are headache and vomiting, as well as weakness, unsteady gait, dizziness, and diplopia [5]. More rarely, patients present with Parinaud syndrome, consisting of double vision, fever, and challenges in speaking [7]. Variants in the *DICER1* gene have been identified as a risk factor for pineoblastoma, and patients with *RB1* mutations have worse outcomes than those lacking the mutations [8]. Age is also considered a risk factor as these tumors are more prevalent in children and adolescents. This case report will focus on an adolescent male who presented to the emergency department and was diagnosed with a pineoblastoma.

## Case presentation

A 16-year-old boy with no significant past medical history presented to the emergency department with headache, neck and back pain, and stiffness for two to three days, accompanied by six episodes of vomiting on the day of presentation. He was taking acetaminophen and ibuprofen without significant relief. He denied fever, upper respiratory symptoms, chest pain, shortness of breath, abdominal pain, dizziness, or weakness. His vaccines were up-to-date and he denied sick contacts or drug use.

On examination, his vital signs were unremarkable apart from an elevated blood pressure of 151/70 mmHg. He was alert and oriented. His pupils were equal and reactive bilaterally with some photophobia. He had decreased extension and flexion of his neck, and both midline and paraspinal cervical tenderness. He had equal strength and sensation bilaterally. The remainder of the examination was unremarkable.

**Figure 1:**
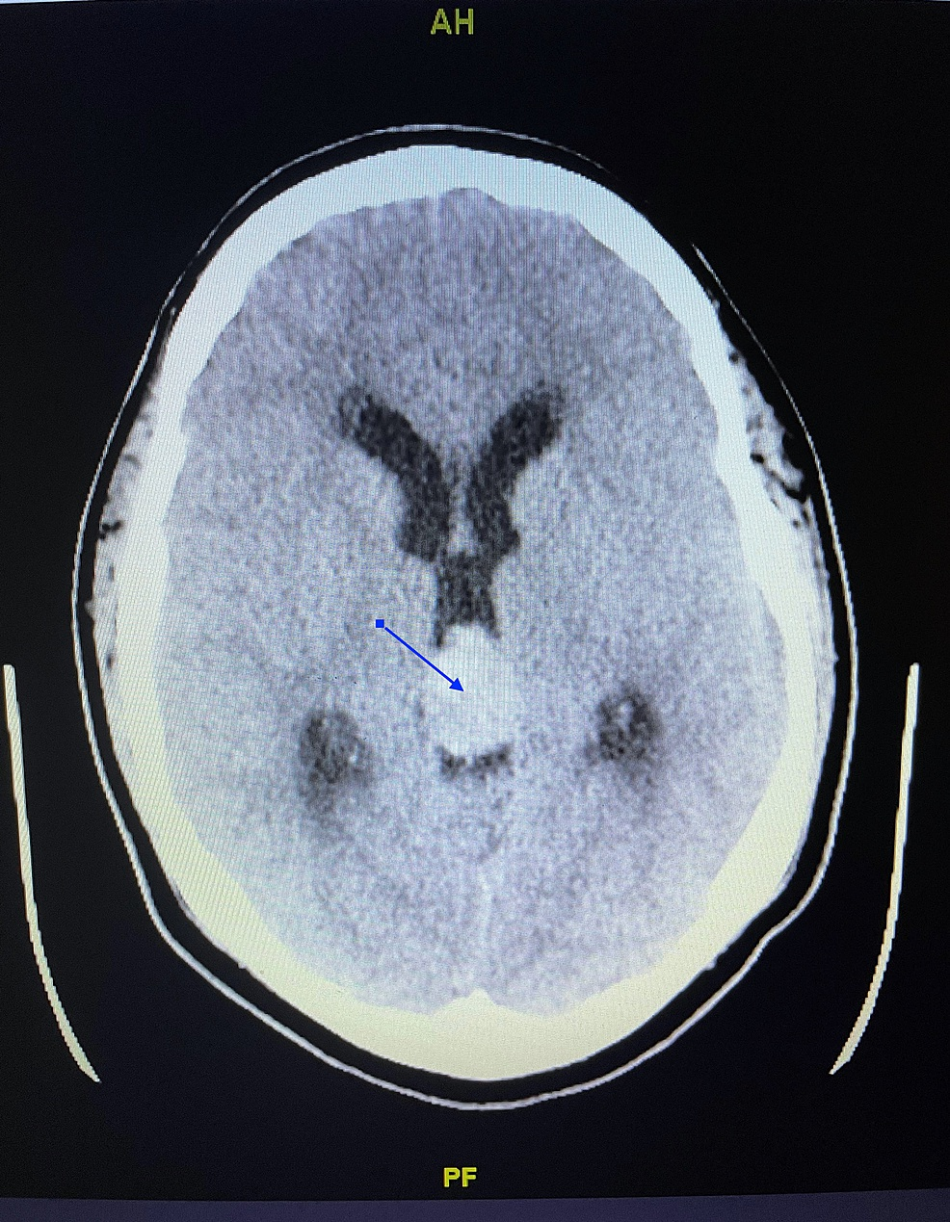
CT scan demonstrating pineoblastoma (arrow). CT: computed tomography

The patient was empirically given ceftriaxone and dexamethasone due to concern for meningitis. Noncontrast brain CT was obtained in preparation for a lumbar puncture to evaluate for meningitis and demonstrated hydrocephalus with transependymal cerebrospinal fluid migration (Figure 1).

An obstructive hyperdense lesion was noted at the level of the third ventricle, likely related to the pineal gland. The patient was transferred to a pediatric hospital where an MRI revealed a pineoblastoma. A ventriculoperitoneal shunt was placed, the tumor was resected, and the patient was started on radiation therapy.

## Discussion

The clinicians on this case conducted a thorough medical history and physical examination. The presence of neck pain, stiffness, and vomiting frequency were causes of concern. The patient was empirically covered for meningitis as a precaution. Performing a noncontrast brain CT during prelumbar puncture protocol was critical to detecting the pineoblastoma. Lumbar puncture to test for meningitis was not performed due to the detection of the mass.

While pineoblastomas are more prevalent in children compared to adults, they remain rare, and the presentation is common to other conditions. This patient exhibited the two most common symptoms, headache and vomiting, along with others associated with the disease. The patient’s age was also a risk factor for pineoblastoma. However, these symptoms and risk factors are by no means indicative of the presence of a pineoblastoma.

**Figure 2:**
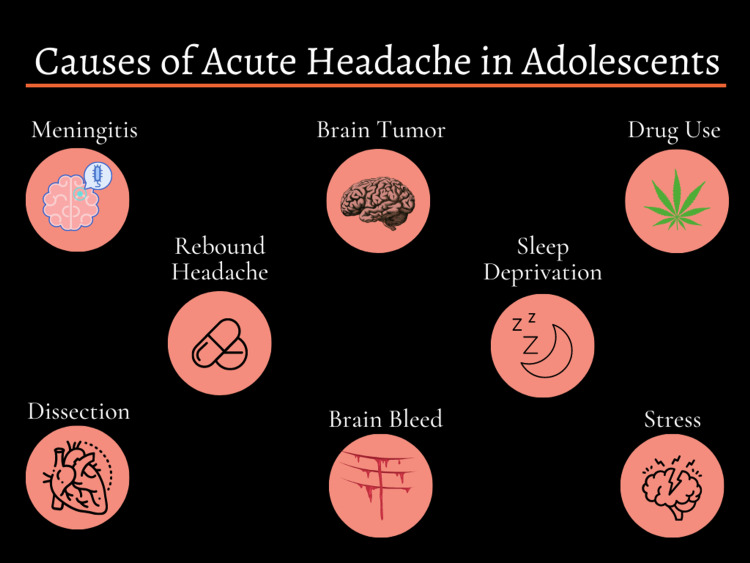
Causes of acute headache in adolescents.

Differential diagnoses in an adolescent male with headache and vomiting symptoms include meningitis, migraine, vertebral or carotid artery dissection, subarachnoid hemorrhage, sleep deprivation, stress, or substance abuse-related conditions such as cannabinoid hyperemesis syndrome (Figure 2).

The patient was precautionarily treated for meningitis, though current vaccination status and lack of fever were suggestive of an alternate etiology. Migraines can also present as headache and vomiting. Approximately 28% of adolescents have migraines, albeit with a female predominance [9]. Repeated vomiting and nausea are also symptoms of cannabinoid hyperemesis syndrome, associated with regular cannabis usage, which has become more prevalent of late [10], and many patients routinely deny drug use. In the United States, by age 16, 29.6% of teens have used marijuana and 14.4% are current users [11]. Because of the frequency of drug use denial and the popularity of marijuana among this age group, it can be tempting to dismiss these symptoms in teens as due to this syndrome. The relative prevalence of these other conditions compared to a pineoblastoma can make it easy to misdiagnose a patient presenting with vomiting and headache. However, a misdiagnosis would lead to late detection of the tumor, which can impact the prognosis.

Numerous factors have been found to influence pineoblastoma prognosis. Patients whose disease has not disseminated at the time of diagnosis have a higher two-year survival rate than those whose disease has disseminated at the time of diagnosis. Other factors that influence prognosis include aggressive surgical resection, chemotherapy, and X-ray therapy, with the most effective being a combination of the three [4]. Studies are inconclusive regarding the impact of tumor size on prognosis. In one study, tumor size greater than 30 mm was associated with a poorer prognosis than tumor size less than 30 mm, though this was not found to be statistically significant [12]. Another study found that measurement of tumor at diagnosis did not impact overall survival [5]. Further research is needed to confirm the impact of these factors. However, the impact of disease dissemination and the possible impact of tumor size on prognosis highlights the importance of an early diagnosis.

## Conclusions

This case could easily have been missed. The patient’s symptoms of headache and vomiting could have been attributed to other, more common, and relatively benign conditions. In the case of this patient, such a misdiagnosis could have greatly delayed tumor diagnosis and treatment, which could have negatively impacted his prognosis. As with many case reports, this one reminds us of the importance of performing a careful history and physical examination and maintaining a high index of suspicion for potentially ominous causes of headache.

## References

[1] Jing Y, Deng W, Zhang H, Jiang Y, Dong Z, Fan F, Sun P (2020). Development and validation of a prognostic nomogram to predict cancer-specific survival in adult patients with pineoblastoma. Front Oncol.

[2] Mynarek M, Pizer B, Dufour C (2017). Evaluation of age-dependent treatment strategies for children and young adults with pineoblastoma: analysis of pooled European Society for Paediatric Oncology (SIOP-E) and US Head Start data. Neuro Oncol.

[3] Mena H, Rushing EJ, Ribas JL, Delahunt B, McCarthy WF (1995). Tumors of pineal parenchymal cells: a correlation of histological features, including nucleolar organizer regions, with survival in 35 cases. Hum Pathol.

[4] Tate M, Sughrue ME, Rutkowski MJ (2012). The long-term postsurgical prognosis of patients with pineoblastoma. Cancer.

[5] Huo XL, Wang B, Zhang GJ (2020). Adverse factors of treatment response and overall survival in pediatric and adult patients with pineoblastoma. Cancer Manag Res.

[6] Kang JK, Jeun SS, Hong YK, Park CK, Son BC, Lee IW, Kim MC (1998). Experience with pineal region tumors. Childs Nerv Syst.

[7] Tian Y, Liu R, Qin J, Wang J, Ma Z, Gong J, Li C (2018). Retrospective analysis of the clinical characteristics, therapeutic aspects, and prognostic factors of 18 cases of childhood pineoblastoma. World Neurosurg.

[8] Schultz KA, Williams GM, Kamihara J (2018). DICER1 and associated conditions: identification of at-risk individuals and recommended surveillance strategies. Clin Cancer Res.

[9] Split W, Neuman W (1999). Epidemiology of migraine among students from randomly selected secondary schools in Lodz. Headache.

[10] Sorensen CJ, DeSanto K, Borgelt L, Phillips KT, Monte AA (2017). Cannabinoid hyperemesis syndrome: diagnosis, pathophysiology, and treatment-a systematic review. J Med Toxicol.

[11] Chen X, Yu B, Lasopa SO, Cottler LB (2017). Current patterns of marijuana use initiation by age among US adolescents and emerging adults: implications for intervention. Am J Drug Alcohol Abuse.

[12] Deng X, Yang Z, Zhang X (2018). Prognosis of pediatric patients with pineoblastoma: a SEER analysis 1990-2013. World Neurosurg.

